# Favipiravir at high doses has potent antiviral activity in SARS-CoV-2−infected hamsters, whereas hydroxychloroquine lacks activity

**DOI:** 10.1073/pnas.2014441117

**Published:** 2020-10-09

**Authors:** Suzanne J. F. Kaptein, Sofie Jacobs, Lana Langendries, Laura Seldeslachts, Sebastiaan ter Horst, Laurens Liesenborghs, Bart Hens, Valentijn Vergote, Elisabeth Heylen, Karine Barthelemy, Elke Maas, Carolien De Keyzer, Lindsey Bervoets, Jasper Rymenants, Tina Van Buyten, Xin Zhang, Rana Abdelnabi, Juanita Pang, Rachel Williams, Hendrik Jan Thibaut, Kai Dallmeier, Robbert Boudewijns, Jens Wouters, Patrick Augustijns, Nick Verougstraete, Christopher Cawthorne, Judith Breuer, Caroline Solas, Birgit Weynand, Pieter Annaert, Isabel Spriet, Greetje Vande Velde, Johan Neyts, Joana Rocha-Pereira, Leen Delang

**Affiliations:** ^a^Laboratory of Virology and Chemotherapy, Department of Microbiology, Immunology and Transplantation, Rega Institute for Medical Research, Katholieke Universiteit Leuven, B-3000 Leuven, Belgium;; ^b^Biomedical MRI and Molecular Small Animal Imaging Centre, Department of Imaging and Pathology, Katholieke Universiteit Leuven, B-3000 Leuven, Belgium;; ^c^Drug Delivery & Disposition, Department of Pharmaceutical and Pharmacological Sciences, Katholieke Universiteit Leuven, 3000 Leuven, Belgium;; ^d^Unité des Virus Emergents, Aix Marseille University, Institut de Recherche pour le Développement (IRD) 190, Institut National de la Santé et de la Recherche Médicale (INSERM) 1207, 13005 Marseille, France;; ^e^UCL Great Ormond Street Institute of Child Health, University College London, WC1N 1EH London, United Kingdom;; ^f^ Molecular Small Animal Imaging Centre, Department of Imaging and Pathology, Katholieke Universiteit Leuven, B-3000 Leuven, Belgium;; ^g^Department of Laboratory Medicine, Ghent University Hospital, 9000 Ghent, Belgium;; ^h^Nuclear Medicine and Molecular Imaging, Department of Imaging and Pathology, Katholieke Universiteit Leuven, B-3000 Leuven, Belgium;; ^i^Assistance Publique–Hôpitaux de Marseille, Aix-Marseille University, Unité des Virus Emergents, Institut de Recherche pour le Développement (IRD) 190, Institut National de la Santé et de la Recherche Médicale (INSERM) 1207, Laboratoire de Pharmacocinétique et Toxicologie, 13005 Marseille, France;; ^j^Translational Cell and Tissue Research, Department of Imaging and Pathology, Katholieke Universiteit Leuven, B-3000 Leuven, Belgium;; ^k^Pharmacy Department, University Hospitals Leuven, 3000 Leuven, Belgium;; ^l^Department of Pharmaceutical and Pharmacological Sciences, Katholieke Universiteit Leuven–University of Leuven, 3000 Leuven, Belgium;; ^m^Global Virus Network, Baltimore, MD 21201

**Keywords:** antiviral therapy, SARS-CoV-2, preclinical model, favipiravir, hydroxychloroquine

## Abstract

Severe acute respiratory syndrome coronavirus 2 (SARS-CoV-2) rapidly spread around the globe after its emergence in Wuhan in December 2019. With no specific therapeutic and prophylactic options available, the virus has infected millions of people of which more than half a million succumbed to the viral disease, COVID-19. The urgent need for an effective treatment together with a lack of small animal infection models has led to clinical trials using repurposed drugs without preclinical evidence of their in vivo efficacy. We established an infection model in Syrian hamsters to evaluate the efficacy of small molecules on both infection and transmission. Treatment of SARS-CoV-2−infected hamsters with a low dose of favipiravir or hydroxychloroquine with(out) azithromycin resulted in, respectively, a mild or no reduction in virus levels. However, high doses of favipiravir significantly reduced infectious virus titers in the lungs and markedly improved lung histopathology. Moreover, a high dose of favipiravir decreased virus transmission by direct contact, whereas hydroxychloroquine failed as prophylaxis. Pharmacokinetic modeling of hydroxychloroquine suggested that the total lung exposure to the drug did not cause the failure. Our data on hydroxychloroquine (together with previous reports in macaques and ferrets) thus provide no scientific basis for the use of this drug in COVID-19 patients. In contrast, the results with favipiravir demonstrate that an antiviral drug at nontoxic doses exhibits a marked protective effect against SARS-CoV-2 in a small animal model. Clinical studies are required to assess whether a similar antiviral effect is achievable in humans without toxic effects.

## Significance

The previous lack of consensus around the use of hydroxychloroquine for COVID-19 patients underlines the need to thoroughly assess the in vivo efficacy of drugs against SARS-CoV-2. Small animal infection models, such as the hamster model, have a pivotal place herein. We here show in vivo preclinical results with favipiravir which indicate that antiviral efficacy against SARS-CoV-2 might only be achieved with a very high dose. Hydroxychloroquine, on the other hand, completely lacks antiviral activity, thus providing no scientific basis for its further use in COVID-19 patients. With this study on two key antiviral candidates, we establish the baseline for SARS-CoV-2 antiviral treatment, which will allow us to identify superior antiviral candidates in the near future.

The severe acute respiratory syndrome coronavirus 2 (SARS-CoV-2) first emerged in Wuhan, China, in December 2019 (1). From there, the virus rapidly spread around the globe, infecting more than 21 million people so far (August 17) https://covid19.who.int/). SARS-CoV-2 is the causative agent of COVID-19. Common clinical manifestations of COVID-19 are fever, dry cough, paired in a minority of patients with difficult breathing, muscle and/or joint pain, headache/dizziness, decreased sense of taste and smell, diarrhea, and nausea (2). A small subset of patients will develop to acute respiratory distress syndrome, characterized by difficult breathing and low blood oxygen levels, which may directly result in respiratory failure (2). In addition, an overreaction of the host’s immune and inflammatory responses can result in a vast release of cytokines (“cytokine storm”), inducing sepsis and multiorgan damage, which may lead to organ failure (3). To date, more than 750,000 patients worldwide have already succumbed to COVID-19. Hence, in response to the ongoing pandemic, there is a desperate need for therapeutic and prophylactic options.

At present, no specific antiviral drugs have been developed and approved to treat infections with human coronaviruses. Nonetheless, antiviral drugs could fulfill an important role in the treatment of COVID-19 patients. Slowing down the replication of SARS-CoV-2 by antiviral treatment could be beneficial and prevent or alleviate symptoms. In addition, antiviral drugs could be used as prophylaxis to protect health care workers and high-risk groups. However, a specific, highly potent antiviral drug for SARS-CoV-2 will take years to develop and evaluate in clinical studies. Therefore, the main focus for COVID-19 treatment on the short term is on the repurposing of drugs that have been approved for other diseases (4). Repurposed drugs can, however, not be expected to be highly potent inhibitors of SARS-CoV-2, since these were not developed and optimized specifically against this virus. In cell culture, several repurposed drugs inhibit SARS-CoV-2 replication (5, 6). Although preclinical in vivo evidence evaluating the efficacy of some of these repurposed drugs for COVID-19 treatment is lacking, clinical trials have already been conducted or are currently ongoing. Two such drugs are hydroxychloroquine (HCQ) and favipiravir.

HCQ is an antimalaria drug that has been widely used to treat patients with malaria, rheumatoid arthritis, and systemic lupus erythematosus. This drug is also able to inhibit a broad range of viruses from different virus families in cell culture, including coronaviruses (SARS-CoV-1, Middle East respiratory syndrome-CoV) (7, 8). Favipiravir is a broad-spectrum antiviral drug that has been approved in Japan since 2014 to treat pandemic influenza virus infections (9). Both drugs have shown antiviral efficacy against SARS-CoV-2 in Vero E6 cells (10), albeit modestly for favipiravir (10–12). Enzymatic assays with the SARS-CoV-2 RNA-dependent RNA polymerase demonstrated that favipiravir acts as a nucleotide analog via a combination of chain termination, slowed viral RNA synthesis, and lethal mutagenesis (12). However, proof of efficacy in animal models is still lacking for both drugs. Nevertheless, clinical trials were initiated early on in the pandemic to assess the efficacy of HCQ and favipiravir to treat COVID-19 patients. For HCQ, these trials were small anecdotal studies or inconclusive small randomized trials (4) and thus did not lead to conclusive results. Despite the lack of clear evidence, HCQ has been widely used for the treatment of COVID-19, often in combination with a second-generation macrolide such as azithromycin. Results from animal models and rigorous randomized controlled trials are thus required to clarify the efficacy of HCQ and favipiravir in the treatment of COVID-19 patients.

Infection models in small animals are crucial for the evaluation and development of antiviral drugs. Although rhesus and cynomolgus macaques seem to be relevant models for studying the early stages of COVID-19 in humans (13), preclinical models using smaller animals are essential to ensure efficient and ethical allocation of resources toward designing (relevant) preclinical and clinical efficacy studies. Syrian hamsters are permissive to SARS-CoV-2 and develop mild lung disease similar to the disease observed in early-stage COVID-19 patients (14, 15). Nevertheless, evidence of antiviral efficacy of repurposed drugs in small animal models is lacking to date. In this work, we characterize Syrian hamsters as a model for the evaluation of antiviral drugs in therapeutic and prophylactic settings against SARS-CoV-2. We then use this model to evaluate the antiviral efficacy of HCQ and favipiravir against SARS-CoV-2 in infected hamsters and in a transmission setting.

## Results

### Characterization of Hamster Model for Antiviral Drug Evaluation.

**Fig. 1.**
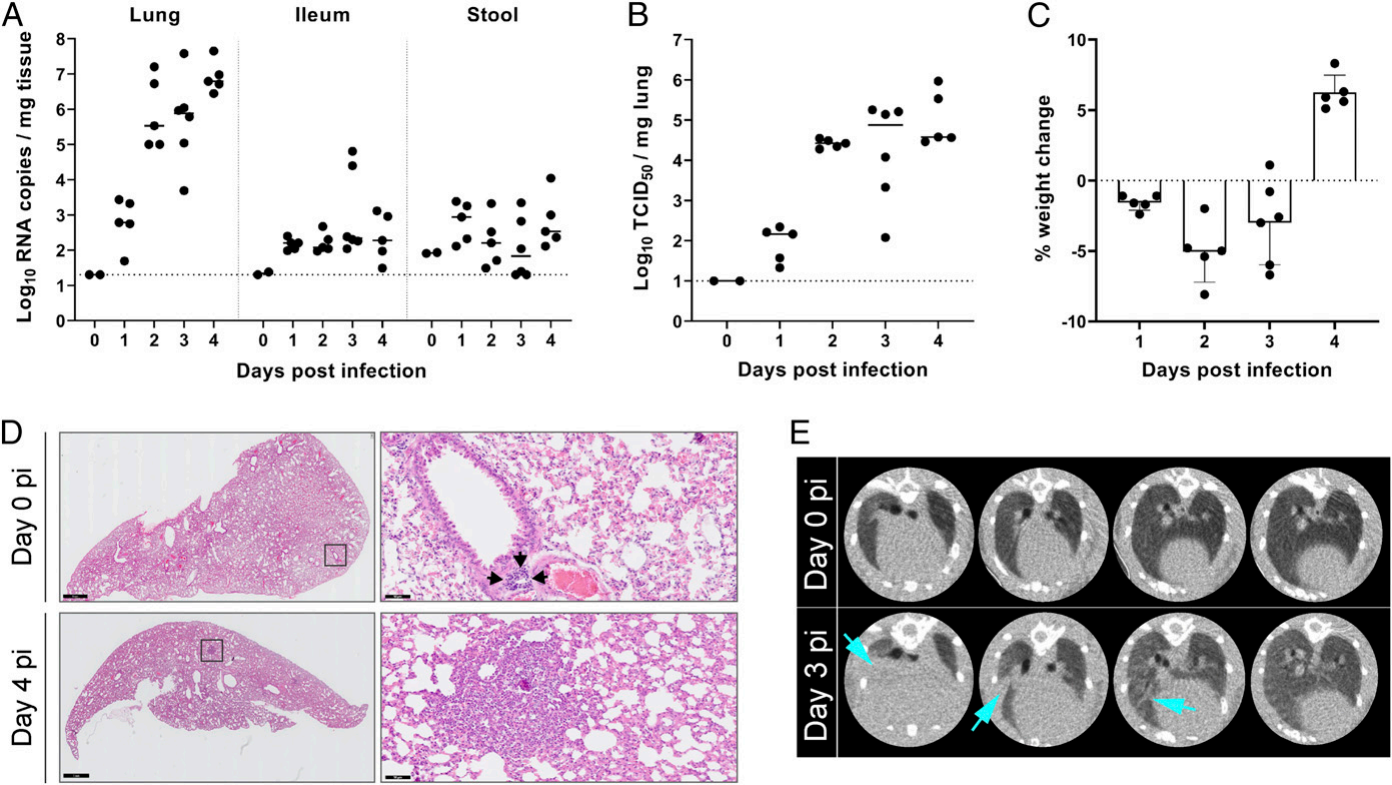
Kinetics of SARS-CoV-2 replication and lung disease in hamsters. (*A*) Viral RNA levels in the lungs, ileum, and stool of infected Syrian hamsters. At the indicated time intervals pi, viral RNA levels were quantified by RT-qPCR. The bars represent median values. (*B*) Infectious viral load in the lung expressed as TCID_50_ per milligram of lung tissue obtained at day 4 pi. The bars represent median values. (*C*) Weight change as compared to the weight at day 0 in percentage at the indicated time intervals pi. Bars represent means ± SD. (*A*–*C*) The data shown are medians plus the individual hamsters represented as separate data points. (*D*) Representative H&E images of lungs of SARS-CoV-2−infected hamsters at day 0 and day 4 pi. At day 0 (*Top*), lungs appear normal; black arrows point at a small lymphoid follicle. At day 4 (*Bottom*), lungs show peribronchial inflammation and bronchopneumonia in the surrounding alveoli. *Right Bottom* shows a small focus of bronchopneumonia; alveolar lumina surrounding a small bronchus are filled with inflammatory cells. Images on the *Right* are magnifications of the black boxes shown in the images on the *Left*. (Scale bars, 1 mm [*Left*] and 50 µm [*Right*].) (*E*) Representative transversal lung micro-CT images of SARS-CoV-2−infected hamsters at baseline (day 0 pi) and day 3 pi. Light blue arrows indicate infiltration by consolidation of lung parenchyma.

We further characterize SARS-CoV-2 infection and readouts of disease in hamsters to be able to use this model for the evaluation and development of antiviral drugs. To investigate SARS-CoV-2 replication and shedding, the lung, ileum, and stool of infected hamsters were harvested at different time points postinfection (pi) for viral RNA quantification by RT-qPCR. Infectious virus titers were additionally determined in lung samples. SARS-CoV-2 efficiently replicates in the lungs of the hamsters, with viral RNA being detected in the lungs from day 1 pi and reaching a maximum level of ∼7 log_10_ RNA copies per mg of tissue at 4 d pi (Fig. 1*A*). A similar kinetic profile was found in the ileum and stool samples, albeit at lower levels of 2 to 3 log_10_ RNA copies per mg of tissue. Titrations of homogenized lung tissue contained infectious particles from 1 d pi and reached levels of ∼5 log_10_ 50% tissue culture infectious dose (TCID_50_)/mg tissue from day 2 pi onward (Fig. 1*B*), which is in line with the viral RNA levels. Infected animals displayed a slight weight loss of about 5% by day 2 pi, which was completely resolved by day 4 pi (Fig. 1*C*). No other signs of disease or distress were observed in the hamsters at any time point pi.

Akin to what is currently done in clinical practice, we evaluated the development of lung disease in a noninvasive way by microcomputed tomography (micro-CT) scanning the infected animals under isoflurane gas anesthesia (16). Dense lung infiltrations and bronchial dilation were simultaneously present from day 3 pi onward, becoming more pronounced at day 4 pi. Longitudinal follow-up of radiological pathology showed signs of multifocal pulmonary infiltrates and lung consolidation on day 3 pi (Fig. 1*D*). Analysis by hematoxylin/eosin (H&E) staining of lungs of infected hamsters at day 4 pi showed signs of bronchopneumonia and peribronchial inflammation, which were not present at the day of inoculation (Fig. 1*E*).

### Evaluation of In Vivo Efficacy of HCQ and Favipiravir.

**Fig. 2.**
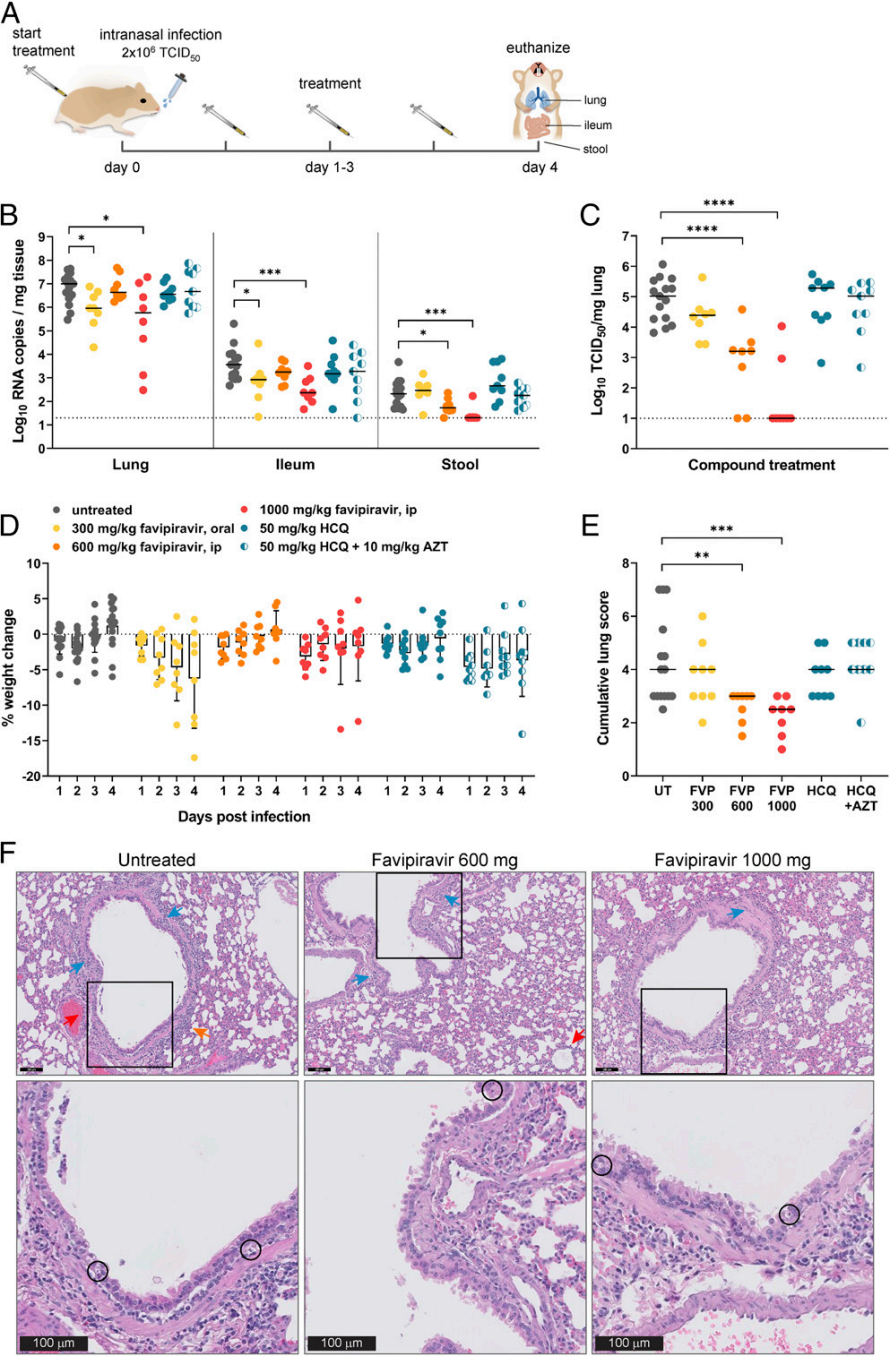
In vivo testing of favipiravir and HCQ in the SARS-CoV-2 infection model. (*A*) Setup of the study. (*B*) Viral RNA levels in the lungs, ileum, and stool of untreated and treated (favipiravir, HCQ, or HCQ + azithromycin) SARS-CoV-2−infected hamsters at day 4 pi. At the indicated time intervals pi, viral RNA levels were quantified by RT-qPCR. The bars represent median values. (*C*) Infectious viral load in the lung of untreated hamsters and hamsters receiving treatment (favipiravir, HCQ, or HCQ + azithromycin) expressed as TCID_50_ per milligram of lung tissue obtained at day 4 pi. The bars represent median values. (*D*) Weight change of individual hamsters (dots) as compared to the weight at day 0 in percentage points at the indicated time intervals pi. Bars represent means ± SD. (*E*) Cumulative severity score from H&E stained slides of lungs from SARS-CoV-2−infected hamsters that were untreated (UT, gray) or treated with favipiravir 300 mg/kg (FVP 300, yellow), 600 mg/kg (FVP 600, orange), 1,000 mg/kg (FVP 1000, red), HCQ (blue), or HCQ + azithromycin (blue-white). The bars represent median values. (*F*) Representative H&E images of lungs at day 4 pi of SARS-CoV-2−infected hamsters and treated with favipiravir. At day 4, lungs of untreated hamsters show significant inflammation in bronchial wall (blue arrows) with apoptotic bodies in respiratory epithelium and extension into adjacent alveoli (orange arrow) and inflammatory cells in arterial wall (red arrow). Lungs of infected hamsters treated with 600 mg⋅kg^−1^⋅d^−1^ favipiravir still show a few inflammatory cells in the bronchial wall (blue arrows) and a few focal perivascular lymphocytes (red arrow), whereas lungs of hamsters treated with 1,000 mg⋅kg^−1^⋅d^−1^ favipiravir contain even less inflammatory cells in the bronchial wall, but no perivascular inflammation. Apoptotic bodies (black circles) were present in bronchial walls of hamsters from all three groups (Scale bars, 100 µm.) Data were analyzed with the Mann−Whitney *U* test. *P < 0.05; **P < 0.01; ***P < 0.001; ****P < 0.0001.

Next, we treated hamsters with antiviral molecules for four consecutive days starting 1 h before intranasal infection with SARS-CoV-2. At day 4 pi, a micro-CT scan was performed, after which the animals were euthanized and organs were collected for quantification of viral RNA, infectious virus titers, and lung histopathology (Fig. 2*A*). Twice-daily treatments for 4 d with favipiravir were given either orally at 300 mg⋅kg^−1^⋅d^−1^ (loading dose of 600 mg⋅kg^−1^⋅d^−1^ on day 0 pi), or by intraperitoneal (i.p.) injection with 600 or 1,000 mg⋅kg^−1^⋅d^−1^ (loading dose of 900 and 1,200 mg⋅kg^−1^⋅d^−1^, respectively, at day 0 pi). Hamsters treated with the low dose of favipiravir presented a decrease of 1.0 log_10_ RNA copies per mg of lung tissue (*P* < 0.05), compared to untreated infected hamsters (Fig. 2*B*); a smaller effect was observed in the ileum and stool of treated animals (Fig. 2*B*). Treatment with the highest dose of favipiravir resulted in similar reductions in viral RNA levels in the lungs, whereas the medium dose had a smaller inhibitory effect (Fig. 2*B*). However, infectious virus titers in the lungs were significantly reduced upon favipiravir treatment in a dose-dependent manner (Fig. 2*C*). While the low dose of favipiravir yielded a reduction of 0.6 log_10_ TCID_50_ per mg in the lungs, the medium and high doses of favipiravir reduced the infectious virus lung titers by 1.8 and 4.0 log_10_ TCID_50_ per mg (*P* < 0.0001 for both), respectively (Fig. 2*C*). In the highest dose group (*n* = 8), only two hamsters had infectious virus in the lungs.

As the reductions in viral infectious titers were more pronounced than those for viral RNA, we calculated the relative lung viral particle infectivity, that is, the ratio between the number of infectious virus particles and the number of viral RNA genomes (*SI Appendix*, Fig. S1*A*). The low dose of favipiravir did not decrease relative infectivity, while the medium and high doses significantly reduced the relative infectivity of lung virus particles by 136- and 172-fold, respectively. Treatment with favipiravir has been shown to drive increased viral mutagenesis leading to defective genomes and loss of infectivity in small animal models and also in patients (17–19). To test whether this was also the case in SARS-CoV-2−infected hamsters, we used Illumina deep sequencing to determine the virus variants in lung samples after favipiravir treatment. The data showed a 3.2-fold increase in mean variants for favipiravir-treated hamsters compared with untreated hamsters (*SI Appendix*, Fig. S1*B*).

Oral treatment with 300 mg/kg favipiravir caused over 5% weight loss at days 3 and 4 pi, which is slightly more than that of the untreated animals (Fig. 2*D*). This could be due to the effect of administering a relatively high volume of compound per os (which was at the limit of 10 mL⋅kg^−1^⋅d^−1^) or due to some toxicity of the molecule. On the other hand, i.p. administrations of favipiravir were better tolerated, with the high dose of 1,000 mg/kg resulting in only 2% weight loss, on average, at day 4 pi. No toxicity signs were observed in the clinical presentation of the hamsters treated with these high doses. Importantly, significant improvements of histological lung pathology were observed in hamsters treated with the medium and high doses of favipiravir (Fig. 2*E*). Peribronchial and perivascular inflammation was focal and less pronounced with the medium dose and nearly absent with the highest dose. The same observation was made with regard to bronchopneumonia (Fig. 2*F*). Quantification of the micro-CT−derived biomarkers of lung pathology measured a relatively small burden of radiological consolidation upon infection that did not change with a low dose and slightly improved with medium and high doses of favipiravir treatment (nonaerated lung volume). Furthermore, no marked differences were observed in the micro-CT−derived markers that measure hyperinflation, emphysema, or atelectasis (*SI Appendix*, Fig. S2).

HCQ sulfate was tested alone or in combination with azithromycin at a dose of 50 mg⋅kg^−1^⋅d^−1^ (equivalent to 39 mg/kg HCQ base) administered i.p. once daily. When in combination, azithromycin was given orally once daily at a dose of 10 mg⋅kg^−1^⋅d^−1^. Treatment with HCQ alone resulted in a very modest reduction of 0.3 log_10_ viral RNA copies per mg of lung, and no reduction in viral RNA load in the ileum or stool, compared to untreated infected hamsters (Fig. 2*B*). When combined with azithromycin, no additional reduction of viral RNA was observed in the organs of infected animals (Fig. 2*B*). Virus titrations of the lungs also revealed no significant reduction after treatment with HCQ alone or in combination with azithromycin (Fig. 2*C*). The weight loss of the animals treated with HCQ follows along the lines of the untreated animals with < 5% weight loss during the whole experiment, while the combination treatment with azithromycin caused a slightly greater weight loss at days 1 and 2 pi, from which the animals could partially recover (Fig. 2*D*). Similarly, no radiological improvement was observed between nontreated animals and animals treated with HCQ or HCQ in combination with azithromycin, which was confirmed by quantification of micro-CT−derived biomarkers of lung pathology (*SI Appendix*, Fig. S2).

### A High Dose of Favipiravir Reduces SARS-CoV-2 Infection in a Transmission Model.

SARS-CoV-2 is typically transmitted through direct contact with respiratory droplets of an infected person or from touching eyes, nose, or mouth after touching virus-contaminated surfaces. Transmission of SARS-CoV-2 through aerosols and direct contact has also been demonstrated in a Syrian hamster model (14, 20). We additionally explored whether SARS-CoV-2 can be transmitted via the fecal−oral route. To this end, hamsters that were intranasally inoculated with virus were euthanized at day 1 or day 3 pi. Subsequently, sentinel hamsters were housed in the used cages of the index hamsters (food grids and water bottles were replaced by fresh ones) and euthanized at day 4 postexposure. Although viral RNA and infectious virus could readily be detected in tissues from index hamsters (except in two stool samples), the majority of sentinel hamsters did not become infected, as shown by the absence of viral RNA and infectious virus in lung and ileum (*SI Appendix*, Fig. S3). This indicates that the fecal−oral route only marginally contributes to the transmission SARS-CoV-2 between hamsters, thereby confirming the results of a previous study (20). We therefore continued by focusing on transmission of the virus via direct contact only.

**Fig. 3.**
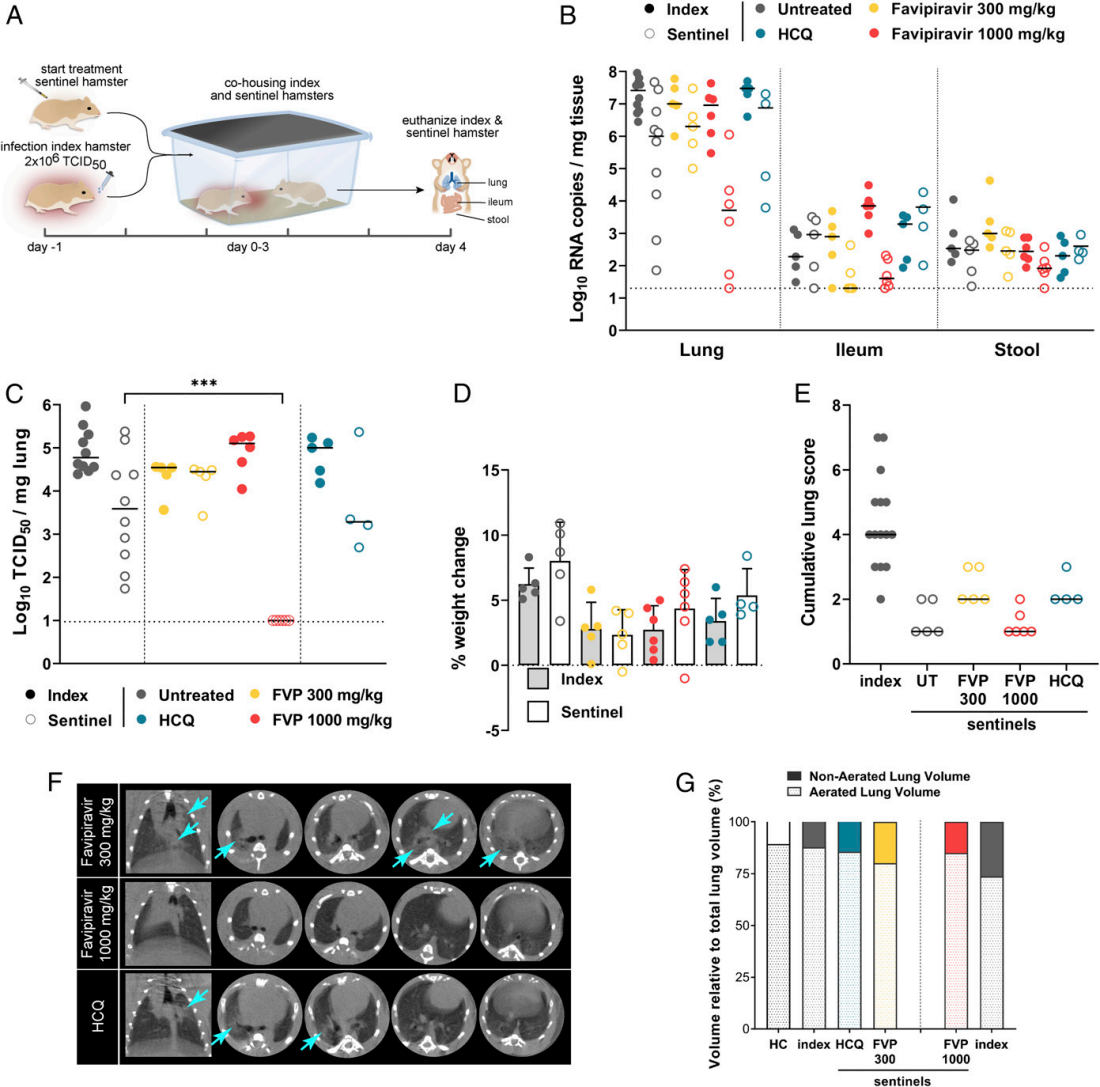
High dose of favipiravir reduces infection in a direct contact transmission model. (*A*) Setup of the study. (*B*) Viral RNA levels in the lungs, ileum, and stool at day 4 pi are expressed as log_10_ RNA copies per milligram of tissue. Closed dots represent data from index hamsters (*n* = 5) inoculated with SARS-CoV-2 1 d before cohousing with sentinel animals. Open dots represent data from sentinel hamsters (*n* = 5 per condition) which were untreated (gray) or treated with either HCQ (blue) or favipiravir 300 mg/kg (yellow), 600 mg/kg (orange), or 1,000 mg/kg (red), starting 1 d before exposure to index animals. The bars represent median values. (*C*) Infectious viral loads in the lung at day 4 pi/postexposure are expressed as log_10_ TCID_50_ per milligram of lung tissue. The bars represent median values. (*D*) Weight change at day 4 pi in percentage, normalized to the body weight at the day of infection (index) or exposure (sentinel). Bars represent means ± SD. (*E*) Cumulative severity score from H&E stained slides of lungs from index SARS-CoV-2−infected hamsters and untreated, favipiravir, and HCQ treated sentinel hamsters. The bars represent median values. (*F*) Representative coronal and transversal lung micro-CT images of favipiravir and HCQ treated sentinel hamsters at day 4 pi. Light blue arrows indicate examples of pulmonary infiltrates seen as consolidation of lung parenchyma. (*G*) Micro-CT−derived nonaerated lung volume (reflecting the tissue lesion volume) and aerated lung volume relative to total lung volume of index SARS-CoV-2−infected hamsters and untreated, favipiravir (FVP), and HCQ treated sentinel hamsters. HC, healthy controls. Data were analyzed with the Mann−Whitney *U* test. ***P < 0.001.

Using the transmission model, we investigated the prophylactic potential of HCQ and favipiravir against SARS-CoV-2. Sentinel hamsters received a daily dosage for five consecutive days with either HCQ (50 mg⋅kg^−1^⋅d^−1^) or favipiravir (300 or 1,000 mg⋅kg^−1^⋅d^−1^ with a loading dose of 600 and 1,200 mg⋅kg^−1^⋅d^−1^, respectively, on the first day of treatment), starting 24 h prior to exposure. Each individual sentinel hamster was cohoused with an index hamster that had been intranasally inoculated with SARS-CoV-2 the day before (Fig. 3*A*). Index hamsters were euthanized 4 d pi, and sentinels were euthanized 4 d postexposure, after which the viral loads in lung, ileum, and stool were determined. Index hamsters had ∼7 log_10_ viral RNA copies per mg in the lungs, whereas untreated sentinel hamsters had ∼4 log_10_ viral RNA copies per mg in the lungs (Fig. 3*B*). The variability between individual hamsters in the sentinel groups was more pronounced than in the index groups. No significant reduction in viral RNA or infectious virus titers in lungs was observed in sentinel hamsters treated with the low dose of favipiravir or with HCQ (Fig. 3 *B* and *C*). Also in ileum and stool, the viral RNA levels were not reduced by compound treatment. Sentinels treated with the high dose of favipiravir showed lower viral RNA levels in the lungs (1.6 log_10_ decrease), albeit not significantly (Fig. 3*B*). Importantly, none of the sentinel hamsters treated with the high dose of favipiravir had infectious virus in the lungs (Fig. 3*C*), indicating that prophylactic dosing with an antiviral drug is able to ameliorate virus infection in hamsters by direct contact. In contrast to the index hamsters, sentinel hamsters did not lose weight, but gained around 8% of body weight by day 4 pi. Sentinels treated with HCQ or favipiravir (both at a low dose and a high dose) gained less body weight than untreated sentinels (5%, 2%, and 4%, respectively) (Fig. 3*D*). Histological lung pathology scores were overall low in untreated and treated sentinel hamsters, providing no additional information on the effect (improvement or worsening) of either drug on lung pathology (Fig. 3*E*). However, we did observe an improvement in pulmonary infiltrates and consolidation in sentinel hamsters treated with the high dose of favipiravir, supported by micro-CT−derived biomarkers of lung pathology. In contrast, sentinels treated with the low dose or HCQ did not show a difference in pulmonary infiltrates and consolidations compared to index hamsters (Fig. 3 *F* and *G*).

### Favipiravir Plasma Concentrations.

**Fig. 4.**
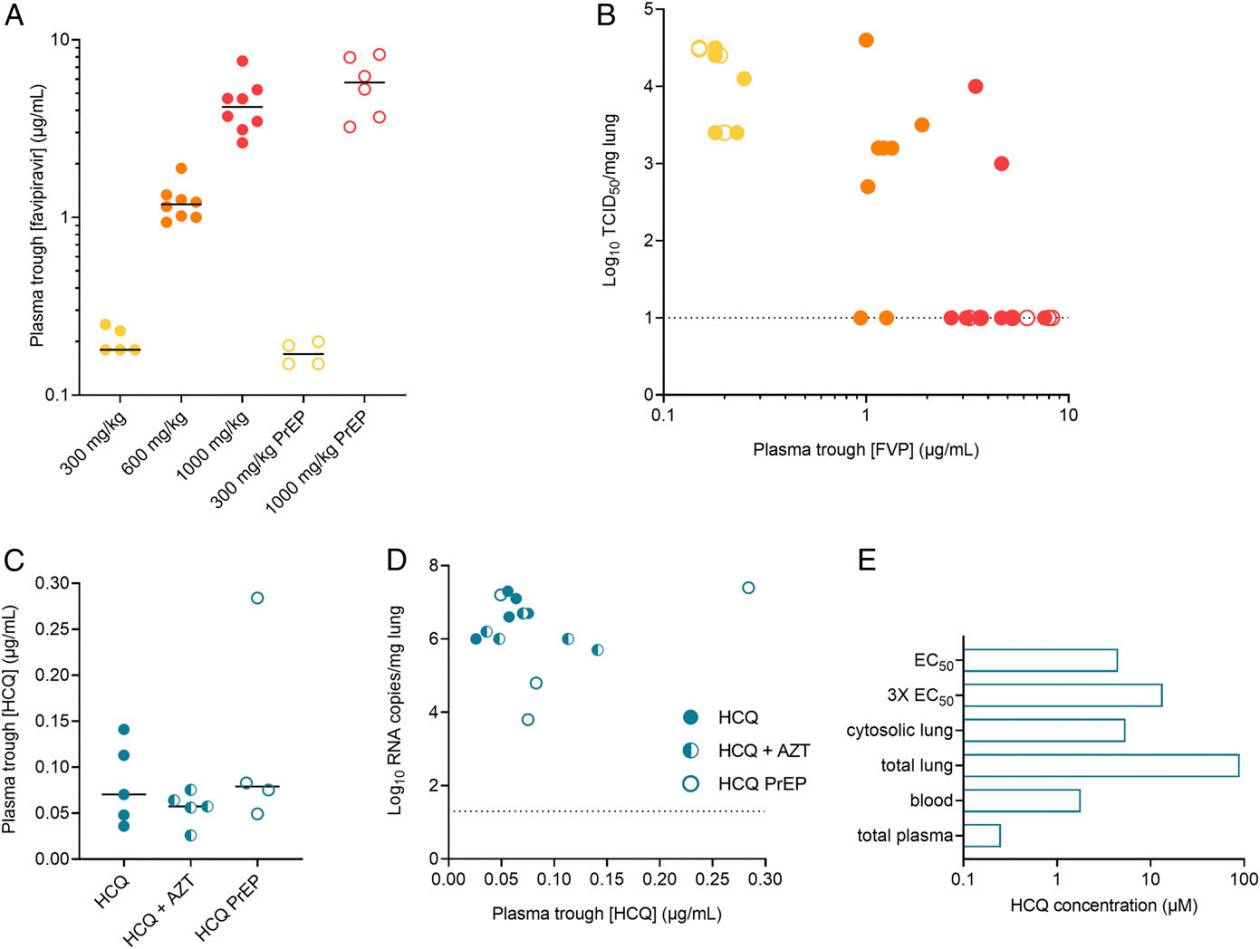
Pharmacokinetics of favipiravir and HCQ in infected and sentinel hamsters. (*A*) Individual plasma trough concentrations of favipiravir in hamsters treated with 300, 600, and 1,000 mg⋅kg^−1^⋅d^−1^ BID. The bars represent median values. PrEP, preexposure prophylaxis (in sentinel hamsters). (*B*) Infectious virus titers in lung tissue at day 4 pi to favipiravir (FVP) plasma trough concentrations of individual hamsters. (*C*) Individual plasma trough concentrations of HCQ in hamsters treated with HCQ or HCQ and azithromycin (AZT) (*n* = 14). The bars represent median values. (*D*) Viral RNA levels in lung tissue at day 4 pi to HCQ plasma trough concentrations of individual hamsters. (*E*) Summary of trough blood and tissue levels of HCQ in hamsters dosed with 50 mg/kg HCQ sulfate and comparison with in vitro EC_50_ values.

To determine the exposure of treated hamsters to favipiravir, plasma trough levels of favipiravir were measured at the time of killing (16 h after the last treatment). As demonstrated earlier in hamsters (17) and nonhuman primates (21), favipiravir showed a nonlinear increase in plasma trough concentrations between different doses after dosing for 4 d (Fig. 4*A*). Oral dosing of 300 mg⋅kg^−1^⋅d^−1^ twice daily (BID) resulted in average trough concentrations of 0.2 ± 0.03 µg/mL, whereas, with i.p. dosing of 600 and 1,000 mg⋅kg^−1^⋅d^−1^ BID, trough levels of 1.2 ± 0.3 µg/mL and 4.4 ± 1.6 µg/mL, respectively, were achieved. Preexposure prophylaxis with 300 and 1,000 mg⋅kg^−1^⋅d^−1^ favipiravir in sentinel hamsters resulted in similar favipiravir trough concentrations after dosing for 5 d. This indicates that the protective effect of the highest favipiravir dose in the transmission model was obtained with an exposure similar to the therapeutic treatment in the infection model. The infectious virus titers in the lung at day 4 pi were significantly associated with favipiravir plasma concentrations in individual hamsters (Fig. 4*B*), indicating that higher exposure to favipiravir resulted in more virus inhibition (*P* < 0.0001, Spearman correlation test, *n* = 31 hamsters).

### Estimation of HCQ Total Lung and Cytosolic Lung Concentrations.

Because we did not observe an effect of HCQ, we performed additional pharmacokinetic (PK) modeling to determine whether this was due to inadequate drug exposure or lack of in vivo efficacy. Based on the measurement of trough concentrations of HCQ at the time of killing (*n* = 14), a mean ± SD trough plasma concentration of 84 ± 65 ng/mL (0.3 ± 0.2 µM) was found (Fig. 4*C*). This is comparable to the plasma trough concentrations that were detected in cynomolgus macaques (treated with a dosing regimen of 90 mg/kg on day 1 pi [loading dose] followed by a daily maintenance dose of 45 mg/kg) (13) and in patients (3 d to 5 d after starting treatment with 200 mg three times daily) (13). The peak viral load in the lungs was not significantly associated with plasma HCQ concentrations in individual hamsters (Fig. 4*D*), suggesting that a higher HCQ exposure did not result in a more pronounced reduction of viral infection (Spearman correlation test, *n* = 14).

According to Eq. **1**, a whole blood concentration of 1.8 ± 1.4 µM was calculated (Fig. 4*E*). Subsequently, applying Eq. **2**, this resulted in a total lung concentration of 90.2 ± 69.4 µM, indicating that the lung tissues achieved HCQ concentrations above the reported in vitro EC_50_ values, ranging from 0.7 µM to 17.3 µM, with a median value of 4.5 µM and an interquartile range of 5.4 (25 to 75%) (22). To estimate 90% of inhibition of viral replication (EC_90_), the EC_90_ was equated to 3 times the EC_50_, resulting in a target lung concentration of 13.5 ± 16.3 µM. In this case, the efficacy target at trough would be reached when applying this dosing regimen (i.e., 50 mg HCQ sulfate per kg/d). However, it is important to note that the total lung tissue concentrations described above consist of both intracellular and interstitial HCQ concentrations. As the in vivo antiviral mechanism(s) of action of HCQ against SARS-CoV-2 has not been clarified yet and might not be exclusively by inhibition of endosome acidification (23), HCQ concentrations were calculated in cytosolic lung tissue, in the endosomal−lysosomal compartment of cells and in the interstitial compartment (Fig. 4*E*). Assuming that cytosolic HCQ concentrations are only 6% of total tissue concentrations, a total cytosolic lung tissue concentration of 5.4 ± 4.2 µM was calculated. This value was in line with the median in vitro EC_50_ value, but is well below the estimated EC_90_ value. Also, the interstitial concentration was calculated to be 5.4 µM. In contrast, the endosomal/lysosomal HCQ concentration was calculated to be 1.9 mM, which is much higher than the estimated EC_90_.

## Discussion

In a previous study, we showed that wild-type Syrian hamsters are highly susceptible to SARS-CoV-2 infections (15). Following on our previous work, here we further characterize the hamster infection model to allow the use of this model for antiviral drug evaluation. In agreement with previous studies, upon intranasal inoculation, we observed that the virus replicates efficiently to peak levels (∼6 log_10_ TCID_50_ per mg) in the lungs on day 4 pi, which is supported by radiological and pathological evidence. Although the virus was also present in the ileum and stool of infected hamsters, levels were significantly lower (∼2.5 log_10_ copies per mg). Besides serving as efficient replication reservoirs of SARS-CoV-2, the hamsters also efficiently transmit the virus to cohoused sentinels (14, 20). Here, we demonstrate that the virus is mainly transmitted via direct contact and only to a limited extent via the fecal−oral route. The variability observed in the virus titers in the lungs of the sentinels is probably due to differences in the infection stage of the animals.

Besides hamsters, a variety of other animals have been tested for their permissiveness to SARS-CoV-2, of which ferrets and nonhuman primates were the most sensitive (24–28). In ferrets, infectious SARS‐CoV‐2 was only detected in the nasal turbinate and, to a lesser extent, in the soft palate and tonsils, but not in the lungs (28). Although infectious virus in the lungs of ferrets was detected in another study, levels remained close to the limit of detection (26). This indicates that ferrets support SARS-CoV-2 replication, albeit to a lesser extent than hamsters. In SARS-CoV-2−infected macaques (both rhesus and cynomolgus), virus levels were the highest in nasal swabs and the lungs (25, 27). SARS-CoV-2 infection resulted in moderate transient disease in rhesus macaques, whereas cynomolgus macaques remained asymptomatic, but did develop lung pathology as seen in COVID-19 (27). Although aged macaque models may represent the best models for studying more severe COVID-19 disease (29), both the high costs and ethical considerations (leading to small group sizes) are major drawbacks of nonhuman primate models. The efficient SARS-CoV-2 replication in the lungs of hamsters combined with development of lung pathology endorses the use of hamsters over any other small animal infection model for preclinical evaluation of the efficacy of antiviral drugs and immune-modulating agents. Potent reduction of SARS-CoV-2 replication in hamsters has been demonstrated by a single dose with a single-domain antibody from a llama immunized with prefusion-stabilized coronavirus spikes (15, 30), thereby validating the use of hamsters to evaluate treatment options against SARS-CoV-2. In addition, our data also indicate that hamsters are highly amenable for studying the potential antiviral effect of small molecules on virus transmissibility in a preexposure and postexposure setting.

In an effort to contribute to the debate on the efficacy of HCQ and favipiravir in COVID-19 patients, we evaluated both repurposed drugs in our hamster infection and transmission model. Treatment with HCQ or combined treatment with azithromycin was not efficacious in significantly lowering viral RNA levels and infectious virus titers in the lungs of SARS-CoV-2−infected hamsters. Lack of efficacy was also demonstrated in the transmission model whereby sentinel hamsters were treated prophylactically prior to exposure to infected hamsters. In SARS-CoV-2−infected ferrets, HCQ treatment was also not able to significantly reduce virus titers (26). In addition, a recent study in SARS-CoV-2−infected cynomolgus macaques showed that HCQ alone or in combination with azithromycin did not result in a significant decrease in viral loads, both in a therapeutic and in a prophylactic setting (13). On the other hand, clinical trials with HCQ for the treatment of COVID-19 patients have resulted in conflicting results and controversy. This is especially the case with clinical studies conducted in the early stage of the pandemic, which were mostly small anecdotal studies. Results of large, placebo-controlled, randomized trials are now becoming available. A randomized trial of HCQ as postexposure prophylaxis after high-to-moderate-risk exposure to COVID-19 showed that high doses of HCQ did not prevent SARS-CoV-2 infection or disease similar to COVID-19 (31). In the Randomised Evaluation of COVID-19 Therapy trial, a large UK-based clinical study to evaluate potential therapies, HCQ treatment did not result in a beneficial effect on mortality or duration of hospital stay in patients admitted with COVID-19 (32). These data are in agreement with our results in the hamster model and clearly underline the importance of preclinical studies in animal models in the drug development/repurposing process.

The lack of effect observed for HCQ in this study and potentially also in other studies may be explained by a PK failure. High lung concentrations of HCQ are caused by massive accumulation (“ion trapping”) of the compound in acidic lysosomes, which is driven by a pH gradient between cytosol (pH 7.2) and lysosomes (pH 5). However, taking into account the pH partition theory and considering the relative volumes of lung cellular and interstitial compartments, only 6% of total HCQ concentration in lung tissue is present in the cytosol of lung cells. The other 94% of HCQ is present in the interstitial compartment and intracellularly in lysosomes/endosomes or other subcellular fractions, or bound to proteins. Starting from the measured trough concentrations from treated hamsters at day 4 or 5, the calculated HCQ concentration in the endosomal compartment was 1.9 mM, which would be well above the EC_90_ target. In contrast, cytosolic concentrations in the lung were only slightly higher than the EC_50_ values reported in the literature, and far below the EC_90_ target. Although alkalization of endosomes has been proposed as one of the key mechanisms of the broad-spectrum antiviral effect of HCQ, the mechanism of action against SARS-CoV-2 has not been completely unraveled (23). Therefore, the very low cytosolic concentrations of HCQ in the lung may explain the absence of an antiviral effect of HCQ against SARS-CoV-2 in vivo. Increasing the HCQ dose to reach the EC_90_ might not be feasible in terms of safety, as it may lead to an increased risk of corrected QT interval prolongation on an electrocardiogram and fatal arrhythmia. In future studies, lung tissue distribution of (repurposed) antiviral drugs should be taken into account, along with specification of the subcellular target site, as recommended by Wang and Chen (33).

In contrast to HCQ, favipiravir inhibited virus infection in intranasally infected hamsters, but only at high doses. Importantly, treatment with high doses also resulted in a significant decrease in lung histopathology. The oral, low dose used in the current study (300 mg⋅kg^−1^⋅d^−1^) was found to be effective in viral infection models using hamsters for, among others, Nipah virus, Rift Valley fever virus, and yellow fever virus (34–36). However, for SARS-CoV-2−infected hamsters, we and others show that this dose is not high enough to markedly inhibit virus infection (17). When analyzing the plasma trough levels of favipiravir upon treatment with this dose, the favipiravir concentrations were, indeed, too low to inhibit SARS-CoV-2 based on in vitro antiviral efficacy. To increase the favipiravir dose, i.p. administration was required, allowing for larger volumes. It was shown previously that i.p. administration of favipiravir resulted in increased plasma concentrations in guinea pigs compared to oral dosing and consequently in improved antiviral efficacy against Junin virus (37). The potent antiviral efficacy of high favipiravir doses is in line with a recent study (17), in which thrice daily (TID) dosing of ∼1,400 mg⋅kg^−1^⋅d^−1^ of favipiravir resulted in significant reductions in virus infection in hamsters. Favipiravir plasma exposures were consistent in both studies, although the doses used were not completely the same. Clinical alleviation of SARS-CoV-2−induced disease (determined by differences in weight loss) was observed. Similar to our findings, the decrease in infectious virus titers was more pronounced than the decrease in viral RNA copies. This discrepancy was shown to be due to the mutagenic effect of favipiravir, as the mean number of mutations in the viral RNA increased by a factor of more than three upon favipiravir treatment. However, in contrast to the lack of toxicity in the highest dose group (1,000 mg⋅kg^−1^⋅d^−1^ BID) in our study, high toxicity was reported with 1,400 mg⋅kg^−1^⋅d^−1^ TID dosing, as evidenced by the significant weight loss from the first day of treatment (17).

In the hamster transmission model, the high dose of favipiravir (1,000 mg⋅kg^−1^⋅d^−1^), given as prophylaxis, markedly blocked viral infection of sentinel hamsters that were in direct contact with infected hamsters. This study thus reports on the ability of an antiviral drug to prophylactically reduce virus infection upon exposure. In agreement with our findings, a high dose of favipiravir (1,400 mg⋅kg^−1^⋅d^−1^ TID) resulted in undetectable viral replication in the lung when treatment was started 1 d before intranasal infection (17).

Clinical trials to evaluate the potency of favipiravir against SARS-CoV-2 are currently ongoing in several countries (38). Previously, an open-label, randomized study already showed that, in COVID-19 patients with mild symptoms (fever and respiratory symptoms without difficulties in breathing), the clinical recovery rate at day 7 was higher in the favipiravir-treated group compared to the control group, which received treatment with arbidol (39). However, for COVID-19 patients with hypertension and/or diabetes as well as critically ill patients, the clinical recovery rate was not significantly different between groups, suggesting that favipiravir might be useful for patients with mild symptoms, but not for severely ill patients. Plasma trough concentrations to potently block virus infection (∼4.4 µg/mL) as measured in the infected hamsters may be achievable in humans treated with favipiravir. In a clinical trial in Ebola virus-infected patients, a favipiravir dosing scheme of 6,000 mg on day 0, followed by 1,200 mg BID for 9 d, resulted in a median plasma trough concentration of 25.9 µg/mL at day 4 (40). This concentration exceeds the plasma concentration measured in hamsters treated with the highest and most effective dose, indicating that an efficacious dose of favipiravir may be achievable in humans. Lung penetration of favipiravir in hamsters has been shown to be efficient, resulting in lung/plasma ratios of 35 to 44% after repeated dosing (17). However, it is not known whether the lung penetration in humans is similar to that in hamsters, limiting a possible extrapolation of our results. On the other hand, it has been reported that the trough concentrations (after 8 h to 12 h) in critically ill COVID-19 patients are lower than those in healthy persons and do not reach the in vitro obtained EC_50_ values against SARS-CoV-2 (41, 42). This unfavorable PK profile of favipiravir has been observed previously in Ebola virus-infected patients (40). Therefore, clinical studies should be conducted to thoroughly evaluate the PK of favipiravir in COVID-19 patients. Another concern remains the safety of favipiravir, as the drug proved to be teratogenic (43). However, favipiravir may be well tolerated and safe with short-term treatment. Nevertheless, potential widespread use of favipiravir for the treatment of COVID-19 patients should be handled with caution.

In conclusion, we here characterize our hamster infection and transmission model to be a robust model for studying the in vivo efficacy of antiviral compounds. Our data endorse the use of Syrian hamsters as the preferred small animal model for preclinical evaluation of treatment options against SARS-CoV-2. In the exceptional situation the world is currently in, clinical trials were initiated at a time when no preclinical data were available. However, at this point, the preclinical data obtained by us and others on HCQ and azithromycin provide no scientific basis for further studies in humans with these molecules. On the other hand, the potent reduction of the viral load in the lungs of hamsters treated with a high dose of favipiravir and its efficacy in the transmission model hint at a potential benefit of this drug in humans. However, clinical studies are needed to confirm whether similar high doses of favipiravir are equally effective and safe in humans. Finally, we emphasize the need to develop highly specific, potent and safe pancorona antiviral drugs. Highly potent drugs are available to treat other viral infections (such as with herpesviruses, HIV, hepatitis B virus, hepatitis C virus, and influenza virus), and it will, without any doubt, be possible, given sufficient efforts, to develop also coronavirus inhibitors. Small animal infection models, such as the hamster model, should have a pivotal place in (de)selecting drugs for clinical development.

## Materials and Methods

### SARS-CoV-2.

The SARS-CoV-2 strain used in this study, BetaCov/Belgium/GHB-03021/2020 (EPI ISL 407976|2020-02-03), was kindly provided by Piet Maes, Katholieke Universiteit Leuven (KU Leuven), Leuven, Belgium. This strain was recovered from a nasopharyngeal swab taken from an RT-qPCR−confirmed asymptomatic patient who returned from Wuhan, China, in the beginning of February 2020 (44). A close relation with the prototypic Wuhan-Hu-1 2019-nCoV (GenBank accession number MN908947.3) strain was confirmed by phylogenetic analysis. Infectious virus was isolated by serial passaging on HuH7 and Vero E6 cells (15); passage 6 virus was used for the studies described here. The titer of the virus stock was determined by end-point dilution on Vero E6 cells by the Reed and Muench method. Live virus-related work was conducted in the high-containment A3 and BSL3^+^ facilities of the KU Leuven Rega Institute (3CAPS) under licenses AMV 30112018 SBB 219 2018 0892 and AMV 23102017 SBB 219 20170589 according to institutional guidelines.

### Cells.

Vero E6 cells (African green monkey kidney, ATCC CRL-1586) were cultured in minimum essential medium (MEM) (Gibco) supplemented with 10% fetal bovine serum (Integro), 1% l-glutamine (Gibco), and 1% bicarbonate (Gibco). End-point titrations were performed with medium containing 2% fetal bovine serum instead of 10%.

### Compounds.

Favipiravir was purchased from BOC Sciences. According to the manufacturer, the purity of favipiravir is ≥97%. HCQ sulfate was acquired from Acros Organics; the purity is 98%. For in vivo treatment, a 30 mg/mL favipiravir suspension was prepared in either 0.4% carboxymethylcellulose (oral gavage) or 3% sodium bicarbonate (i.p. injection), and a 20 mg/mL HCQ sulfate solution was prepared in 10% dimethyl sulfoxide, 18% Cremophor, and 72% water. Azithromycin was provided by the hospital pharmacy of the University Hospitals Leuven, Belgium, as a 40 mg/mL oral solution (Zitromax), which was diluted to 5 mg/mL with an aqueous medium consisting of 0.6% xanthan gum as viscosity enhancer.

### SARS-CoV-2 Infection Model in Hamsters.

The hamster infection model of SARS-CoV-2 has been described before (15). In brief, wild-type Syrian hamsters (*Mesocricetus auratus*) were purchased from Janvier Laboratories and were housed per two in ventilated isolator cages (IsoCage N Biocontainment System, Tecniplast) with ad libitum access to food and water and cage enrichment (wood block). Housing conditions and experimental procedures were approved by the ethical committee of animal experimentation of KU Leuven (License P065-2020).

Female hamsters 6 wk to 10 wk old were anesthetized with ketamine/xylazine/atropine and inoculated intranasally with 50 µL containing 2 × 10^6^ TCID_50_. Drug treatment was initiated 1 h before infection. Favipiravir was administered twice daily for 4 d with 300 (oral gavage), 600, or 1,000 mg⋅kg^−1^⋅d^−1^ (i.p. injection), starting with a loading dose of 600, 900, or 1,200 mg⋅kg^−1^⋅d^−1^, respectively, on the first day. HCQ sulfate (50 mg/kg) was administered once daily by i.p. injection for 4 d. Azithromycin (10 mg/kg) was administered once daily by oral gavage using a 5 mg/mL dilution of Zitromax. Hamsters were daily monitored for appearance, behavior, and weight. At day 4 pi, hamsters were euthanized by i.p. injection of 500 μL of Dolethal (200 mg/mL sodium pentobarbital, Vétoquinol SA), in agreement with the guidelines of the KU Leuven Ethical Committee. Tissues (lungs, small intestine [ileum]) and stool were collected, and viral RNA and infectious virus were quantified by RT-qPCR and end-point virus titration, respectively. Blood samples were collected at day 4 pi for PK analysis of HCQ and favipiravir.

### Deep Sequencing and Analysis of SARS-CoV-2 in Lung Samples.

Genome sequences from all samples were obtained using SureSelectXT target enrichment and Illumina sequencing. Reads generated were trimmed with Trim Galore (45). Duplicated reads were removed using Picard (46). A consensus genome for the inoculated virus was obtained by mapping the reads from the inoculation sample to the SARS-CoV-2 reference genome (NC_045512) from GenBank using the Burrows–Wheeler Alignment tool with maximal exact matches. The mapping quality was checked using Qualimap, and the consensus whole genome sequence for the inoculated virus was generated using quantification and annotation of short reads in R (47, 48). Reads from the hamster lung samples were mapped against the consensus inoculate sequence to identify variants. Poor-quality genomes with less than 90% coverage and less than a 100-mean read depth were excluded (four out of eight cases, zero out of six controls). Variants occurring at >1% with a minimum of four independent supporting reads per strand were identified using VarScan (49).

### SARS-CoV-2 Transmission Model in Hamsters.

The hamster transmission model of SARS-CoV-2 via direct contact has been described previously (14, 20). Briefly, index hamsters (6 wk to 10 wk old) were infected as described above. At the day of exposure, sentinel hamsters were cohoused with index hamsters that had been intranasally inoculated with SARS-CoV-2 1 d earlier. Index and sentinel hamsters were euthanized at day 4 pi (postexposure in the case of the sentinels), and the viral load in lung, ileum, and stool was determined, as described above. For prophylactic testing of drugs, sentinel hamsters were treated daily for five consecutive days with either HCQ (50 mg/kg, once daily) or favipiravir (300 or 1,000 mg⋅kg^−1^⋅d^−1^, twice daily), starting 1 d prior to exposure to the index hamster.

To study the contribution of the fecal−oral route to the overall transmission of SARS-CoV-2, index hamsters were inoculated as described earlier. On day 1 or 3 pi, the index hamsters were euthanized, after which sentinel hamsters were placed in the dirty cages of the index hamsters. Food grids and water bottles were replaced by clean ones to minimize virus transmission via food or water. At day 4 postexposure, the sentinels were euthanized. Tissues (lung, ileum, and stool) were collected from index and sentinel hamsters and processed for detection of viral RNA and infectious virus.

### PK Analysis of Favipiravir in Plasma.

Favipiravir was determined in plasma as described before (17).

### PK Analysis of HCQ and Metabolite in Plasma.

HCQ and its active metabolite desethylhydroxychloroquine (DHCQ) were quantified in (ethylenedinitrilo)tetraacetic acid−plasma samples. A total of 1) 50 µL of sample and 2) 10 µL of internal standard solution (HCQ-d4 1,500 ng/mL in water) were added to a tube and mixed. After addition of 50 µL of 5% perchloric acid, samples were shaken for 5 min and centrifuged for 5 min at 16,162 × *g*. Then, 5 µL of the supernatant was injected onto the high-pressure liquid chromatography (HPLC) column.

HPLC analysis was performed using a Shimadzu Prominence system (Shimadzu) equipped with a Kinetex C18 column (100 mm length × 2.1 mm inner diameter, 2.6-µm particle size) (Phenomenex) at 50 °C. A 6-min gradient of mobile phase A (0.1% formic acid [FA] in water) and B (0.1% FA in acetonitrile) with a flow rate of 0.4 mL/min was used for elution of the compounds. The mass spectrometer was a Triple Quad 5500 (Sciex) with an electrospray ionization source in positive ion mode, using multiple reaction monitoring. The monitored transitions were 336.8 *m/z* to 248.0 *m*/*z*, 307.8 *m*/*z* to 130.0 *m*/*z*, and 340.8 *m*/*z* to 252.0 *m*/*z* for HCQ, DHCQ, and HCQ-d4, respectively. The used collision energy for all of the transitions was 30 V. Calibration curves for both HCQ (linear 1/*x* weighting) and DHCQ (quadratic 1/*x*^2^ weighting) were between 10 ng/mL and 2,250 ng/mL. Between-run imprecision over all QC levels (10, 25, 400, and 2,000 ng/mL) ranged from 2.84 to 11.4% for HCQ and from 5.19 to 10.2% for DHCQ.

### Calculation of HCQ Concentration in the Lung Cytosol.

Starting from the measured total trough plasma concentrations measured at killing after 4 d or 5 d of HCQ treatment, total lung cytosolic concentrations of HCQ were calculated. First, the mean trough total plasma concentration of HCQ was used as a starting point to estimate the whole blood concentrations considering a blood to plasma ratio of 7.2, as reported by Tett et al. (50) and as mentioned in the summary of product characteristics of Plaquenil (Sanofi) (Eq. **1**).

whole blood concentration=plasma concentration×7.2. [1]

Relying on the experimental Kp (tissue versus whole blood partition coefficient) values in rats, the total lung tissue concentrations of HCQ were determined. Based on the partition values as reported by Wei et al. (51), a lung Kp value of 50 was applied to estimate the total lung concentration (Eq. **2**).

total lung tissue concentration=mean blood concentration×50. [2]

Subsequently, as the HCQ efficacy target is intracellular, the cytosolic/total HCQ concentration ratio was estimated, based on 1) relative lysosomal lung tissue volume, as well as the contributions of interstitial and intracellular volumes to total lung volume, and 2) the pH partition theory applying a pK_a_ value of HCQ of 9.67. Based on these calculations, lung cytosolic HCQ concentrations correspond to 6% of the total lung tissue concentration (Eq. **3**).

total cytosolic lung tissue concentration=total lung tissue concentration×0.06. [3]

The calculated total cytosolic lung concentration was compared with EC_50_ concentrations previously reported in literature, ranging from 0.72 µM to 17.3 µM (52–54).

### SARS-CoV-2 RT-qPCR.

Hamster tissues were collected after killing and were homogenized using bead disruption (Precellys) in 350 µL of RNeasy lysis buffer (RNeasy Mini kit, Qiagen) and centrifuged (10,000 rpm, 5 min) to pellet the cell debris. RNA was extracted according to the manufacturer’s instructions. To extract RNA from serum, the NucleoSpin kit (Macherey-Nagel) was used. Of 50 μL of eluate, 4 μL was used as a template in RT-qPCR reactions. RT-qPCR was performed on a LightCycler96 platform (Roche) using the iTaq Universal Probes One-Step RT-qPCR kit (BioRad) with N2 primers and probes targeting the nucleocapsid (15). Standards of SARS-CoV-2 complementary DNA (Integrated DNA Technologies) were used to express viral genome copies per milligram of tissue or per milliliter of serum.

### End-Point Virus Titrations.

Lung tissues were homogenized using bead disruption (Precellys) in 350 µL of MEM and centrifuged (10,000 rpm, 5 min, 4 °C) to pellet the cell debris. To quantify infectious SARS-CoV-2 particles, end-point titrations were performed on confluent Vero E6 cells in 96-well plates. Viral titers were calculated by the Reed and Muench method using the Lindenbach calculator (55) and were expressed as TCID_50_ per milligram of tissue.

### Histology.

For histological examination, the lungs were fixed overnight in 4% formaldehyde and embedded in paraffin. Tissue sections (5 μm) were analyzed after staining with H&E, and scored blindly for lung damage by an expert pathologist. The scored parameters, to which a cumulative score of 1 to 3 was attributed, were the following: congestion, intraalveolar hemorrhage, apoptotic bodies in bronchial epithelium, necrotizing bronchiolitis, perivascular edema, bronchopneumonia, perivascular inflammation, peribronchial inflammation, and vasculitis.

### Micro-CT and Image Analysis.

Micro-CT data of hamster lungs were acquired in vivo using dedicated small animal micro-CT scanners, either using the X-cube (Molecubes) or the Skyscan 1278 (Bruker Belgium). In brief, hamsters were anesthetized using isoflurane (2 to 3% in oxygen) and installed in prone position into the X-cube scanner using a dedicated imaging bed. A scout view was acquired, and the lung was selected for a nongated, helical CT acquisition using the High-Resolution CT protocol, with the following parameters: 50 kVp, 960 exposures, 32 ms/projection, 350-μA tube current, rotation time 120 s. Data were reconstructed with 100-µm isotropic voxel size using a regularized statistical (iterative) image reconstruction algorithm (56). On the SkyScan1278, hamsters were scanned in supine position under isoflurane anesthesia, and the following scan parameters were used: 55 kVp X-ray source voltage and 500-μA current combined with a composite X-ray filter of 1-mm aluminum, 80-ms exposure time per projection, acquiring four projections per step with 0.7° increments over a total angle of 220°, and 10 cm field of view covering the whole body, producing expiratory weighted three-dimensional data sets with 50-μm isotropic reconstructed voxel size (57). Each scan took ∼3 min.

Visualization and quantification of reconstructed micro-CT data were performed with DataViewer and CTan software (Bruker Belgium). As primary outcome measure, a semiquantitative scoring of micro-CT data was performed as previously described (56). Visual observations were blindly scored (from 0 to 2, depending on severity, both for parenchymal and airway disease) on five different, predefined transversal tomographic sections throughout the entire lung image for both lung and airway disease by two independent observers, and averaged. Scores for the five sections were summed up to obtain a score from 0 to 10, reflecting severity of lung and airway abnormalities compared to scans of healthy, wild-type control hamsters. As secondary measures, imaging-derived biomarkers (nonaerated lung volume, aerated lung volume, total lung volume, and respective densities within these volumes) were quantified as previously (15, 57, 58) on a manually delineated volume of interest covering the lung, avoiding the heart and main blood vessels. The threshold used to distinguish aerated from nonaerated lung volume was manually defined and kept constant for all datasets (57, 58).

### Statistics.

GraphPad Prism (GraphPad Software, Inc.) was used to perform statistical analysis. Statistical significance was determined using the nonparametric Mann−Whitney *U* test. *P* values of ≤ 0.05 were considered significant.

## Supplementary Material

Supplementary File

## Data Availability

All data supporting the findings of this study are available in the main text and/or *SI Appendix*.
