# Supplementary material for: Favipiravir at high doses has potent antiviral activity in SARS-CoV-2−infected hamsters, whereas hydroxychloroquine lacks activity
Source: Proc Natl Acad Sci U S A. 2020 Oct 9;117(43):26955–65. doi: 10.1073/pnas.2014441117 (PMC7604414; doi:10.1073/pnas.2014441117)
Supplement: Supplementary File [file pnas.2014441117.sapp.pdf]

PNAS
www.pnas.org

Supplementary Information for

Favipiravir at high doses has potent antiviral activity in SARS-CoV-2-infected hamsters, whereas hydroxychloroquine lacks activity

Suzanne J. F. Kaptein[a,1], Sofie Jacobs[a,2], Lana Langendries[a,2], Laura Seldeslachts[b,2], Sebastiaan ter Horst[a], Laurens Liesenborghs[a], Bart Hens[c], Valentijn Vergote[a], Elisabeth Heylen[a], Karine Barthelemy[d], Elke Maas[a], Carolien De Keyzer[a], Lindsey Bervoets[a], Jasper Rymenants[a], Tina Van Buyten[a], Xin Zhang[a], Rana Abdelnabi[a], Juanita Pang[e], Rachel Williams[e], Hendrik Jan Thibaut[a], Kai Dallmeier[a], Robbert Boudewijns[a], Jens Wouters[f], Patrick Augustijns[c], Nick Verougstraete[g], Christopher Cawthorne[h], Judith Breuer[e], Caroline Solas[i], Birgit Weynand[j], Pieter Annaert[c], Isabel Spriet[k], Greetje Vande Velde[b], Johan Neyts[a,l,1], Joana Rocha-Pereira[a,1,3], Leen Delang[a,1,3]

Corresponding authors: Suzanne J. F. Kaptein, Johan Neyts, Joana Rocha-Pereira, Leen Delang

Email: suzanne.kaptein@kuleuven.be, johan.neyts@kuleuven.be, joana.rochapereira@kuleuven.be, leen.delang@kuleuven.be

This PDF file includes:

Figures S1 to S3

www.pnas.org/cgi/doi/10.1073/pnas.2014441117

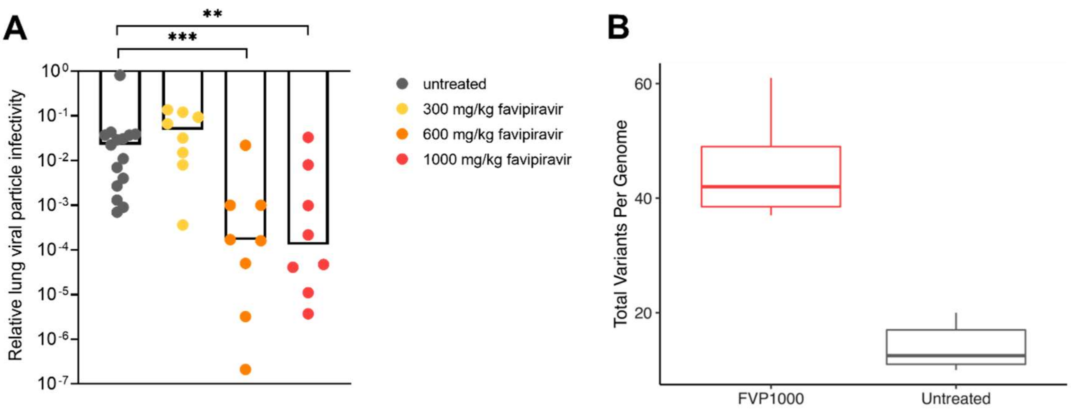

Fig. S1. Relative lung viral particle infectivity and virus variants of SARS-CoV2. (*A*) Relative lung viral particle infectivity was calculated as the ratio of infectious virus particles per mg lung ($TCID_{50}$/mg) over the viral RNA yield per mg/lung (virus copies/mg). Data of individual hamsters are presented as dots; the line represents the median value. Data were analyzed with the Mann-Whitney *U*-test. **, *P* <0.01; ***, *P* <0.001. (*B*) Box plot to show virus variants above 1% in the lungs of favipiravir-treated (1000 mg/kg) and untreated SARS-CoV-2-infected hamsters.

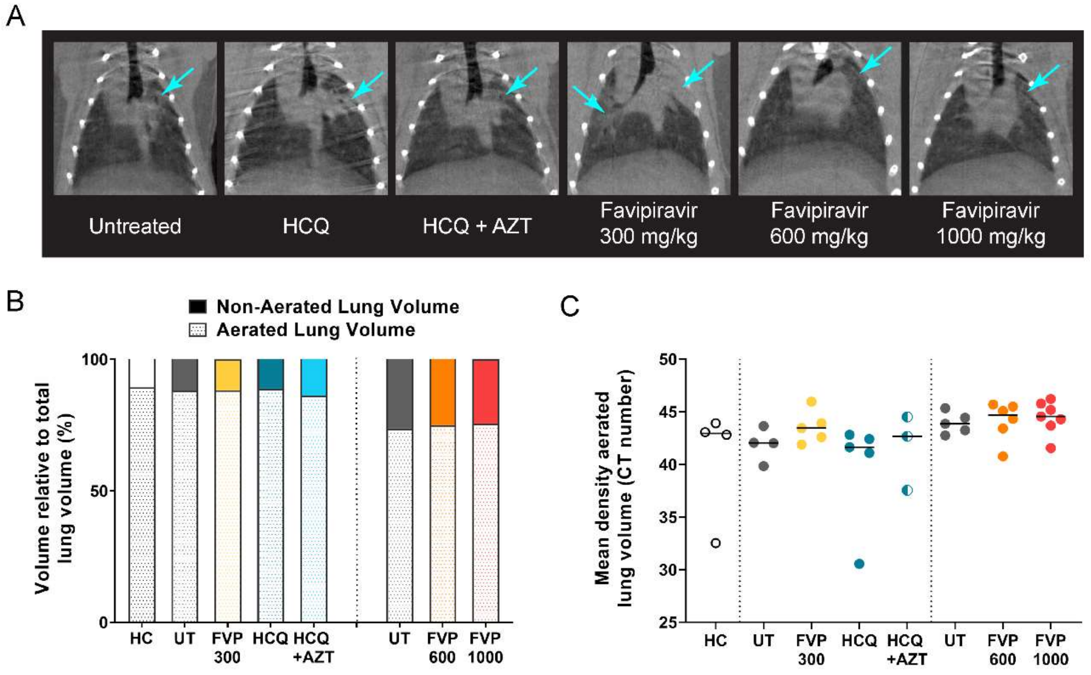

Fig. S2. Micro-CT-derived biomarkers of lung pathology. (*A*) Coronal lung micro-CT images at 4 dpi of SARS-CoV-2-infected hamsters, untreated (UT) or treated with favipiravir (or FVP), HCQ or HCQ + azithromycin (or AZT). Light blue arrows point to examples of pulmonary infiltrates observed as consolidation of lung parenchyma. (*B*, *C*) Quantification of micro-CT-derived biomarkers, as determined in two experiments (separated by a dashed line): non-aerated lung volume (reflecting the tissue lesion volume) and aerated lung volume relative to total lung volume (*B*) and mean density of the aerated lung volume (*C*). HC, healthy controls (white).

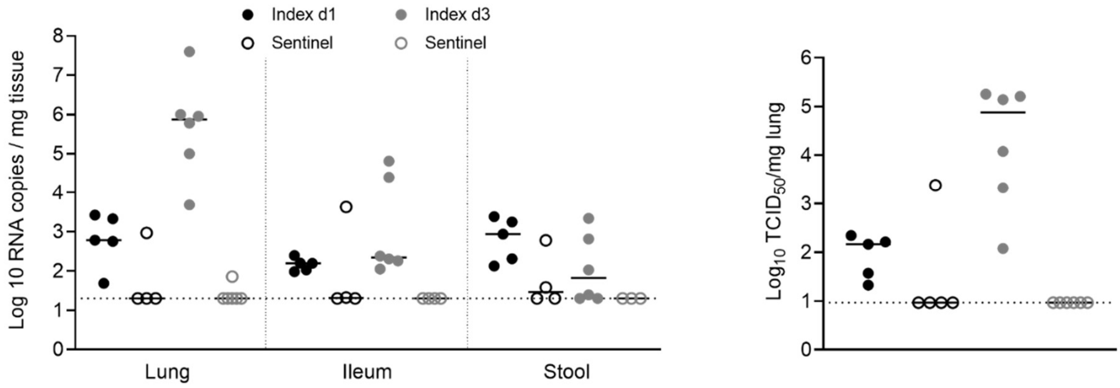

Fig. S3. SARS-CoV-2 transmission by fecal-oral route is not efficient. (*A*)Viral RNA levels in the lungs, ileum and stool of index hamsters at day 1 (black) and day 3 pi (grey), and of sentinel hamsters that were exposed for 4 days to the feces of the index hamsters. Viral RNA levels were quantified by RT-qPCR. (*B*) Infectious viral load in the lung of index hamsters at day 1 (black) and day 3 pi (grey) and sentinel hamsters at day 4 post fecal exposure expressed as $TCID_{50}$ per mg of lung tissue.
